# Characterization of Geometrical Changes of Spherical Advanced Pore Morphology (APM) Foam Elements during Compressive Deformation

**DOI:** 10.3390/ma12071088

**Published:** 2019-04-02

**Authors:** Matej Borovinšek, Matej Vesenjak, Yoshikazu Higa, Ken Shimojima, Zoran Ren

**Affiliations:** 1 Faculty of Mechanical Engineering, University of Maribor, 2000 Maribor, Slovenia; matej.vesenjak@um.si (M.V.); zoran.ren@um.si (Z.R.); 2 Department of Mechanical Systems Engineering, National Institute of Technology (KOSEN), Okinawa College, 905 Henoko, Nago, Okinawa 905-2192, Japan; y.higa@okinawa-ct.ac.jp (Y.H.); k_shimo@okinawa-ct.ac.jp (K.S.)

**Keywords:** APM foam, compression loading, micro computed tomography, porosity analysis, geometrical analysis

## Abstract

The mechanical properties of Advanced Pore Morphology (APM) foam elements depend strongly upon their internal porous and external structural geometry. This paper reports on a detailed investigation of external (e.g. shape and size) and internal (e.g. distribution, size, number of pores) geometry and porosity changes of APM foam elements, during compressive loading by means of the ex-situ micro-Computed Tomography, and advanced digital image analysis and recognition. The results show that the porosity of APM foam elements decreases by only 25% at the engineering strain of 70% due to an increase of the number of pores at high stages of compressive deformation. The APM foam elements also exhibit a positive macroscopic Poisson’s ratio of υ = 0.2, which is uncharacteristic for cellular structures.

## 1. Introduction

**Figure 1:**
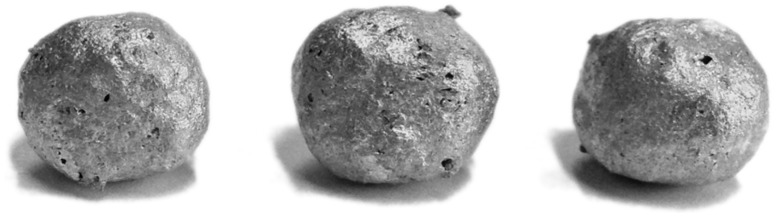
Advanced Pore Morphology (APM) foam specimens 1, 2 and 3.

APM foam is a hybrid cellular material developed at the Fraunhofer Institute IFAM, Bremen, Germany [1,2]. It consists of bonded or un-bonded APM foam elements with an approximately spherical shape, and a thin solid (integral) outer shell, along with a complex closed-cell internal structure (Figure 1) [3,4].

The APM foam elements fabrication process consists of powder compaction (by the CONFORM process) and rolling of AlSi7 alloy with a TiH_2_ foaming agent to obtain expandable precursor material. The precursor material is cut into small pieces, which are then expanded into quasi-spherical foam elements due to heat reaction of the TiH_2_ foaming agent in a continuous belt furnace.

A detailed description of the technology concept and production can be found in [2,5]. The APM foam elements can be manufactured from 3 to 15 mm in diameter, with bulk density varying from 500 to 1000 kg/m^3^ [1]. Single APM foam elements are usually bonded together with a polymer (Polyamide PA12 or Araldite AT 1-1) into an APM structure [5,6]. Such structure exhibits the inner porosity of a single APM foam element of 0.63–0.82, and an outer porosity due to the space between APM foam elements assembly, which varies with different APM foam element sizes in a range of 0.4–0.5 [7,8]. APM foam elements can also be used in APM-based polymer matrix foams, where the entire space between APM foam elements is filled with polymer filler to improve their mechanical response further [9,10]. As for other Metal Matrix Syntactic Foams (MMSF), which were developed from their polymer matrix counterparts, and are studied widely by many authors [11,12,13,14], APM foam elements could also be used as fillers for MMSF.

APM foams have a wide range of possible applications due to their specific mechanical [2,15,16,17] and thermal [18] properties, e.g., as elements for reinforcing hollow structural elements, as deformation elements for absorbing impact energy, for vibration damping in dynamically excited components, and as an internal layer of composite materials [19,20]. One of their main advantages is the ease in filling the hollow components of complex shapes, in order to increase their stiffness and absorption capacity during deformation and collapse. APM foam elements can fill any hollow part easily, regardless of the complex shape of its cavity, which is otherwise impossible using conventional foam materials. APM foam elements have a characteristic stress-strain curve in compression, similar to other cellular materials [17,21,22,23]. Their mechanical properties are described in detail in [2,5,7,15,17], and are also compared to other cellular metals in [20].

For wider application of the APM foam in industrial applications, it is important to predict their behavior correctly in specific applications by means of experimental testing or advanced computational modeling [3,15,18]. The mechanical and thermal properties of APM structures depend strongly on the complex internal geometry of the APM foam elements, as is known for other closed-cell foams [24]. In [25] the authors studied the porosity of individual APM elements and the porosity of the bulk APM structures theoretically. The experimental structural characterization of these APM foam elements is reported in [3]. The authors have used micro-Computed Tomography (μCT) scanning of APM foam elements to obtain the μCT images, which were analyzed to determine the spatial and size distribution of pores. Additionally, the influence of the base materials (AlSi7 and AlSi10) on the internal porous structure, has been studied [26].

This paper analyzes the shape and porosity changes of the APM foam elements during compressive loading, and contributes to a better understanding of their behavior under loading.

## 2. Methods

### 2.1. Micro-Computed Tomography and Ex-Situ Compression

Analysis of shape and porosity changes in the Advanced Pore Morphology (APM) foam elements during compressive loading was observed by use of micro-Computed Tomography. Three different APM specimens (specimens 1, 2 and 3) were analyzed, with a nominal outer diameter of 10 mm, and weighing between 369 mg and 382 mg. The compressive loading was performed (ex-situ) outside the micro-Computed Tomography (μCT) device, and the deformation of the specimens was recorded step-by-step in eight equally distributed stages of engineering strain from 0% to 70%. 

**Figure 2:**
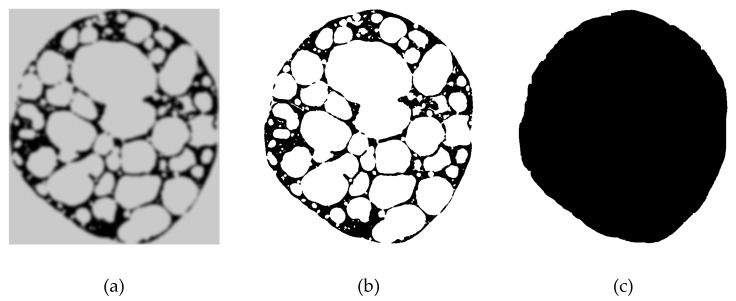
Image analysis displaying one layer (cross-section) of the APM element. (**a**) Grayscale micro-Computed Tomography (μCT) image; (**b**) Segmented black and white image; (**c**) Segmented interior image.

μCT allows for analysis of a material’s interior with an arbitrary complex structure [9]. The μCT investigations reported in this paper were carried out on a Micro CT TOSCANER-30000 Series System machine, manufacturer Toshiba. A voltage of 70–80 kV and a current of 55–60 μA was applied for the data acquisition. 1800 two-dimensional (2D) images (layers) were recorded for each selected specimen, with a physical resolution of 4 μm at a digital resolution of 1024 × 1024 pixels, and spaced 10 μm apart in a vertical direction. The captured data can be represented as grayscale images (Figure 2a), where the color corresponds to the local material density at the observed position. Analysis of the outer shape and porosity was first carried out for each layer separately. 

The results were then combined to build the entire three-dimensional (3D) model of the observed specimen, which was then used for an analysis of the pore size and the pore size distribution.

### 2.2. Geometrical Characterization

The first step in the μCT image analysis is the segmentation, which separates the material domain from the background, based on the selected grayscale threshold color (Figure 2b; black shows the material domain of APM foam material). The color threshold was set in such a way that the volume of the material domain of the computer model was the same as the volume of the base material of the APM foam specimen, which was determined from the mass of the specimen. Using this procedure, the deviation of all computer models was lower than 0.4% in comparison to the volume of the fabricated specimens.

The porosity of the cellular material $p=V_{p}/V_{0}$ is defined as the ratio between the volume of pores $V_{p}$ and the outer volume of the observed cellular material $V_{0}$, where the outer volume of the observed material $V_{0}=V_{p}+V_{m}$ is the sum of the pore volume, and the volume of the base material $V_{m}$.

Only the base material volume can be determined from the segmented images, since the white color on the segmented images represents the pores and the background together (Figure 2b). The background was separated from the pores by using the following automated procedure. Four rays were constructed, from each white pixel of the segmented image to the edge of the image in the following directions: Up, down, left and right. If at least two rays did not intersect any black pixels on their way to the edge of the image, the pixel was treated as a background pixel, and vice versa. The result of this procedure on a single layer is shown in Figure 2c, where the black pixels now represent the interior area of the APM foam element on one layer. The outer volume of the complete APM foam element $V_{0}$ was then computed by multiplying the determined interior area of the APM element on each layer (Figure 2c) with its thickness, and then summing up all of the layer volumes. The volume of pores $V_{p}$ was determined similarly by multiplying the pore area (Figure 2b) inside the interior area of the APM foam element on each layer with its thickness, and then summing together all of the layer volumes. In this way, the porosity of a single layer and the porosity of the whole specimen was determined automatically from digital μCT images.

The change in the APM foam element shape under compressive loading was determined through the average layer radius, which was defined as a radius of the circle with the same area as the area of the APM foam element interior on a layer. The pores inside the APM foam elements were represented by the largest inscribed spheres, to analyze the size of the pores and their distribution in the APM foam element’s volume. A distance transform algorithm [27] was used to determine the corresponding inscribed sphere diameter. The spheres were centered at local maximums of the distance transform field, where “local” refers to the voxels’ Moore Neighborhood [28]. 

Due to geometrically uneven (non-spherical and non-smooth) pore walls in the APM foam elements, this procedure can detect a large number of inscribed spheres in a single pore. Since only one inscribed sphere should be located inside one pore, the detected spheres were merged together if the distance between the sphere centers was smaller than 6/10 of the sum of their radii. The procedure is described in more detail in [3].

The analysis of the APM foam elements’ μCT images was first done on a pixel basis, and then changed to length units (mm) using the resolution of 4 μmpp.

## 3. Results and Discussions

A detailed deformation analysis is given hereafter just for the APM foam element specimen 1, since the other two specimens exhibited very similar behavior. The herein observations are also true for the other two specimens.

**Figure 3:**
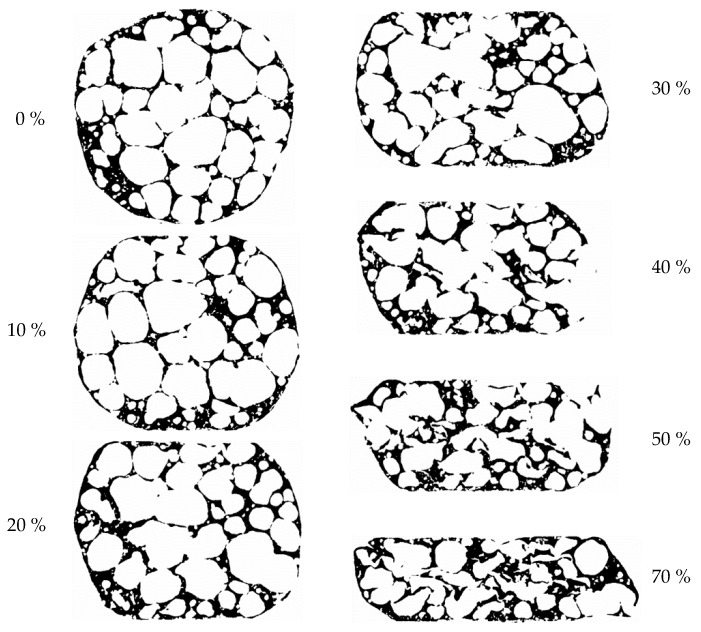
Change of the outer shape and internal structure of APM foam element specimen 1 at compressive engineering strains of 0, 10, 20, 30, 40, 50 and 70%.

The specimen 1 mid-section deformation during compressive loading is shown in Figure 3 for all steps of compressive engineering strain between 0% and 70%. During the ex-situ μCT process of loading and scanning, the specimen was transferred repeatedly to the loading machine and back, with some possible rotation of the specimen around its vertical axis. The sections shown in Figure 3 are, therefore, not directly comparable, so that only quantities independent from the rotation were compared. The Figure indicates that the deformation of the specimen first starts at the contact with the support and load surface. The contact surfaces on the upper and lower contact boundaries are very similar in size in all deformation stages. This indicates a similar local stiffness of specimen 1 at its top and bottom parts.

**Figure 4:**
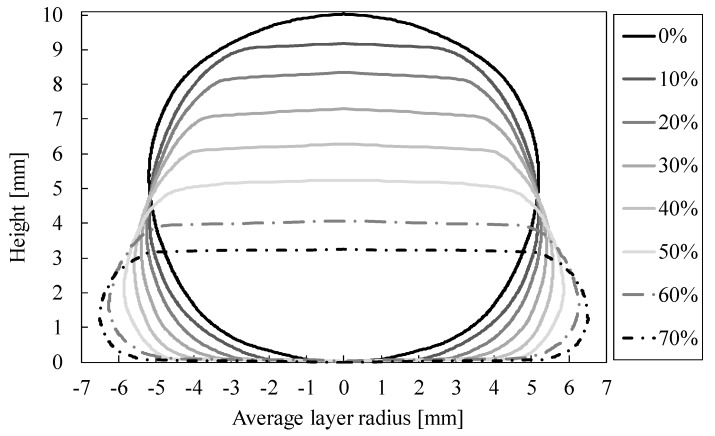
Average layer radius of the APM foam element specimen 1 in relation to the specimen height at different engineering strains.

A more detailed analysis of the external shape change of the specimen was performed by analyzing the average layer radius and the total volume of the specimen in relation to the engineering strain. Figure 4 shows the size of the average layer radius of specimen 1 as a function of the specimen height at different engineering strain stages. 

The Figure indicates that increasing the engineering strain also increases the lateral deformation of the specimen, which indicates that the macroscopic Poisson number of the observed specimen is positive.

**Figure 5:**
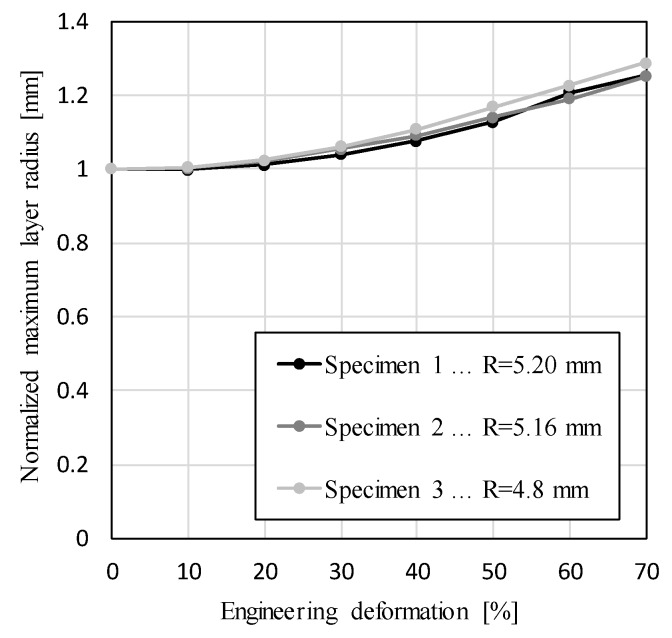
Normalized maximum average layer radius.

Figure 5 shows the change of the maximum average layer radius of all three specimens during their compression. The values of the maximum layer radius were normalized by their initial size, which was equal to 5.20 mm, 5.16 mm and 4.8 mm for specimens 1, 2 and 3, respectively. Comparison of results between the specimens shows very similar behavior, where the size of the maximum layer radius first increases very slowly, and then changes more rapidly in an almost linear fashion. The slow increase of the maximum layer radius at the first stages of the compression is the result of local specimen deformation at the supporting surfaces. Since this deformation is limited to the sample parts in contact with the supporting surfaces, it has no effect on the sample deformation at the maximum layer cross-section. As the compression progresses, the deformation zone increases to a larger portion of the sample, changing the maximum layer radius. The largest relative change in the maximum layer radius equals 28.7% at the last stage of the deformation of specimen 3, while the smallest relative change equals 25.1%, and is observed in specimen 2. The relative difference between the largest and smallest maximum layer radius of the specimens at the largest deformation stage equals 12.5%.

**Figure 6:**
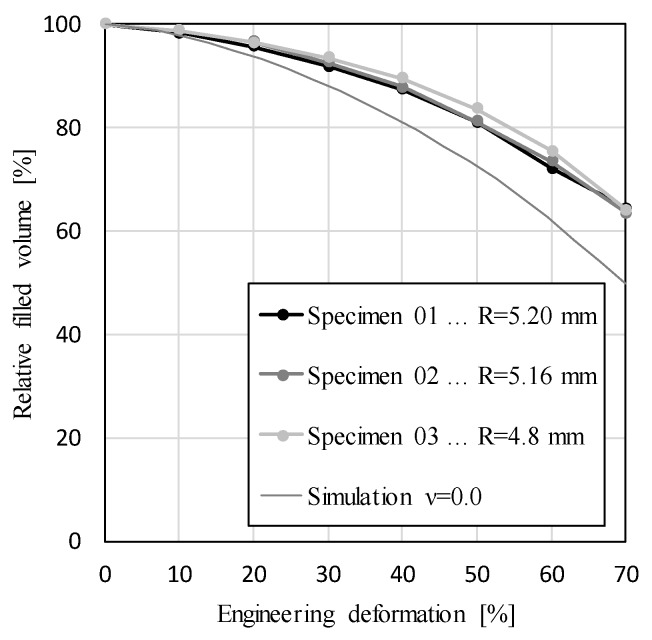
Filled APM foam element volume.

However, the filled specimen volume decreases through deformation stages with an increasing rate, which is shown in Figure 6. The maximum relative change of the filled volume in comparison to the initial volume equals 36.5% at the last deformation stage of specimen 3, while the lowest equals 35.6% in specimen 1. The relative difference between individual specimens is even smaller than the difference in the maximum layer radiuses, and is equal to 2.5%. This small difference indicates that filled specimen volume depends mostly on global APM foam sample properties like outer shape and porosity, which do not differ much between individual APM foam samples, and not on local sample properties like size, shape and position of the pores.

Figure 6 also shows the change in volume of the perfect sphere with linear elastic material properties and Poisson ratio of υ = 0.0, which was determined by the computer simulation using the Finite Element Method (FEM). The same procedure was used to determine the equivalent Poisson’s ratio of analyzed APM foam element specimens inversely, which returned the value υ = 0.2, which best represents the measured experimental results.

The relationship between the specimen volume and the engineering strain can be approximated by a second order polynomial:

(1) $$\frac{V}{V_{0}}=A\varepsilon^{2}+B\varepsilon +C,$$

where $V_{0}$ represents the initial volume of the specimen, V and ε the current volume and engineering strain, respectively, and A, B and C are constants of the polynomial. The constants were determined according to all three specimens simultaneously, so that the coefficient of determination $R^{2}$ was minimal. The following polynomial constants were retrieved: $A=3.30\cdot 10^{-1}$, $B=2.83\cdot 10^{-1}$ and C=1.03, with the coefficient of determination equal to $R^{2}=0.997$.

**Figure 7:**
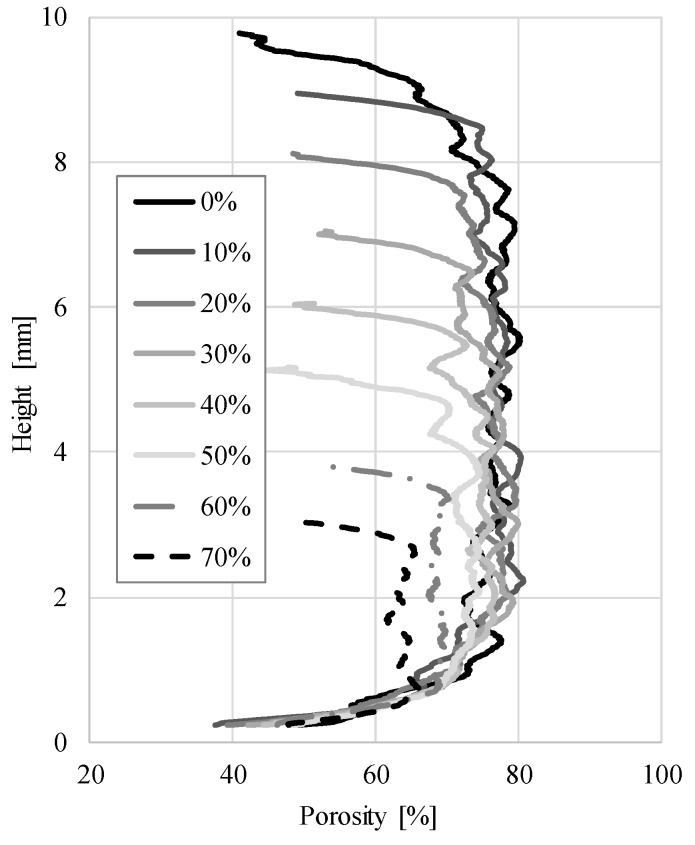
The specimen 1 porosity change through specimen height.

The porosity change through the specimen height in relation to the engineering strain is shown in Figure 7. It can be seen that the porosity varies through the specimen height. The porosity is highest and almost constant through the middle bulk of the specimen (about 74% in average). Constant porosity in the middle of the samples indicates that pores are evenly distributed inside the specimens and that there are no significant deviations, such as large pores or lack of pores, which would change the porosity of the sample cross-section. At the top and bottom of the specimen the porosity drops quickly at all stages of the deformation. 

**Figure 8:**
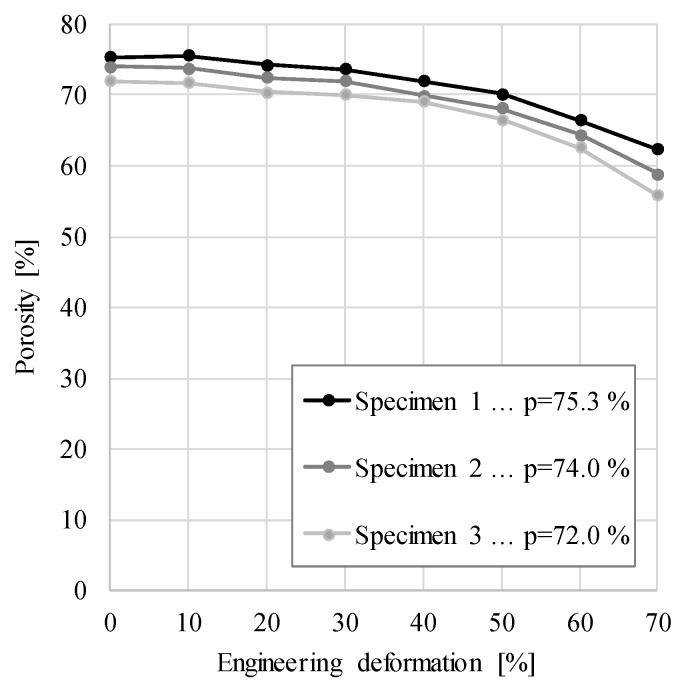
The average specimen porosity change under compressive loading.

This is due to the almost solid outer skin of the APM foam element. As expected, the porosity decreases with increasing compressive deformation, but only to an average porosity of about 60% at maximum 70% strain. The porosity change at different engineering strains for all specimens is shown in Figure 8. The largest relative change in porosity was measured in specimen 3, where the porosity changed from 72.0% to 55.9% (Δ_3_ = 22.3%), while the smallest difference occurred in specimen 1, where the porosity decreased from 75.3% to 62.4% (Δ_1_ = 17.2%). In specimen 2 the porosity decreased from 74.0% to 58.8% (Δ_2_ = 20.5%). Despite the applied engineering strain of 70%, the porosity of APM foam elements decreased by less than 25% during deformation.

**Figure 9:**
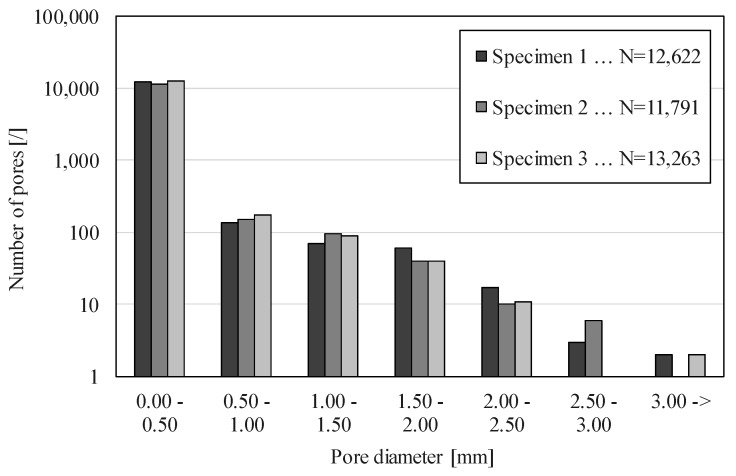
Pore size distribution in APM foam element specimens before the compression.

**Figure 10:**
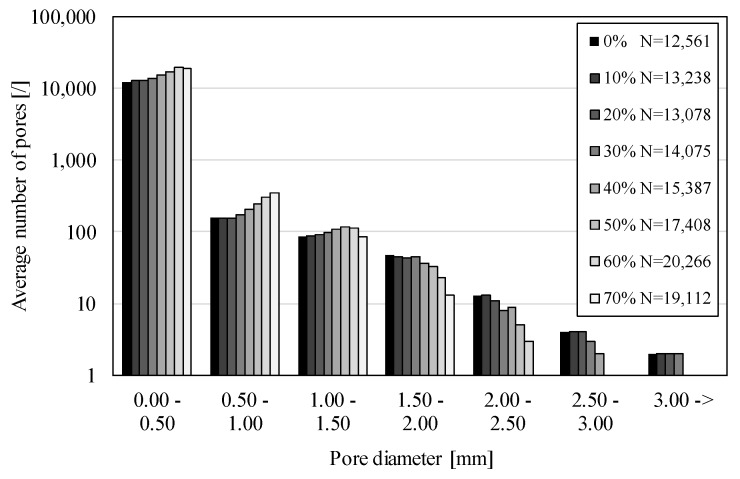
Average pore size distribution in APM foam element specimens during deformation.

The pore size distribution before and during the compressive deformation is shown in Figure 9 and Figure 10. Figure 9 shows pore size distribution individually for all three specimens. The largest number of pores N = 13,263, was found in specimen 3, and the smallest number of pores was equal to N = 11,791 in specimen 2. 

Comparing these values with the porosity of individual specimens shows that the number of pores is not a good indicator for specimen porosity, since specimen 3, which has the largest number of pores, has the smallest porosity.

The pore sizes were divided into six equal intervals of 0.5 mm in the range from 0 to 3 mm, and an additional interval for pores larger than 3 mm, to evaluate the pore size distribution (Figure 10). A very large number of pores was found in the smallest interval from 0 to 0.5 mm, where the size of the smallest detected pore depended upon the μCT resolution of the 3D image (4 μm). This result agrees with the previous studies [3], where the existence of numerous micro-pores was mentioned for the first time. The largest detected pore had a diameter of 3.2 mm.

Comparing the number of pores in different pore size intervals shows a rapid decrease in pore number as the pore size increases (Figure 9 and Figure 10 use the logarithmic scale). The first interval contains approximately 10,000 pores per specimen, while the second interval contains only about 150 pores per specimen. The number of pores decreases to only 1 and 2 pores in the largest pores size interval.

Differences between specimens in pore size distribution are small considering the random process of pores formation. The relative difference between the specimens with the smallest and largest number of pores in the first interval equals $\Delta_{r}$ = 11.2%. The two largest intervals contain less than 10 pores, which is not enough to be statistically representable, so the largest relative difference was determined for the pore size interval from 2 to 2.5 mm, and equals $\Delta_{r}$ = 41.2%.

Figure 10 shows the change in average pore size distribution in all APM foam element specimens during compressive deformation. Surprisingly, the average total number of pores rises from 12,561 to 19,112 pores, which is an increase of 52.2%. The first three smallest pore size intervals show an increase in the pore number as the deformation increases. This increase is the largest in the first interval, where the number of pores changes from 12,254 to 18,658. From the fourth to the last pore size interval the number of pores decreases as the deformation increases. This result indicates that, first, the larger pores in the interval from 1.5 to 3 mm deform and collapse through local wall buckling during the APM foam element deformation. This causes self-contact of pore walls, thus effectively dividing them into smaller pores.

## 4. Conclusions

The shape and porosity change analysis of APM foam elements during the ex-situ compressive loading was done by means of micro-Computed Tomography and digital image analysis. Three APM foam element specimens with an external diameter of 10 mm were analyzed at a step-wise increase of engineering compressive strain between 0% and 70%. The analysis results provide new insights into the deformation behavior of the APM foam elements during compressive loading.

The volume of the specimens decreased during the compressive loading, while their outer diameter increased. The maximum decrease in initial volume of the specimens was only 36.5% at 70% of engineering deformation. The inverse computational simulations of these specimens’ compressive testing revealed the macroscopic Poisson’s ratio of APM foam elements to be equal to 0.2, which is uncharacteristically high for porous material.

The porosity of APM foam elements changes during deformation. The analysis results showed an even distribution of porosity before and after the deformation in the middle bulk part of the specimens, while the porosity dropped quickly towards the top and bottom parts of the specimens due to denser outer skin. The porosity of APM foam elements before the deformation was approximately 74%, and it reduced only by 25% to approximately 58% at the engineering strain of 70%. 

The pore size and pore size distribution analysis showed that APM foam specimens are similar in internal structure, and contain a large number of very small pores, while the number of larger pores is low. The number of larger pores decreases during deformation, as larger pores collapse and form new smaller size pores.
